# Oral Health in Hospitalized Adults: Clinical Outcomes and the Role of Dental Expertise

**DOI:** 10.7759/cureus.116067

**Published:** 2026-09-10

**Authors:** Kevin Benites Chuman

**Affiliations:** 1 Dentistry, Universidad Nacional Federico Villarreal, Lima, PER

**Keywords:** hospital-acquired pneumonia, hospital dentistry, hospitalized adults, interdisciplinary care, nursing oral care, oral health, oral hygiene, dental professionals

## Abstract

Oral health may receive limited attention during hospitalization despite the high burden of oral disease, functional dependency, and reduced ability to maintain routine oral hygiene among medically complex patients. Multiple observational studies report associations between impaired oral health and broader clinical vulnerability or adverse outcomes, including functional impairment, infectious complications, prolonged hospitalization, and mortality. At the same time, hospital oral-health management ranges from patient self-care and routine nursing-led hygiene to structured oral-care programs and direct treatment by dental professionals, yet the relative contributions of these approaches remain unclear.

This narrative review synthesizes current evidence on the relationship between oral-health status and clinical outcomes in hospitalized adults, evaluates the potential impact of oral-health interventions, and examines the role of dental professionals within interdisciplinary hospital care. PubMed and Scopus were searched in August 2026 for literature published from 2015 through 2026 using complementary strategies addressing oral-health status, respiratory and broader clinical outcomes, oral-care interventions, and dental or interdisciplinary involvement. Earlier foundational studies were retained when directly relevant to the review question.

The strongest intervention evidence concerns respiratory outcomes, with systematic reviews and randomized trials suggesting benefits from structured oral care and mechanical plaque removal in selected hospitalized populations. Recent randomized evidence provides preliminary support for structured multicomponent oral care in reducing non-ventilator hospital-acquired pneumonia in general hospital wards. However, benefits have not been demonstrated consistently across all clinical outcomes, and evidence regarding specific components, such as chlorhexidine, remains inconsistent. Professional dental involvement may provide additional value through assessment, diagnosis, treatment of active oral disease, staff education, protocol development, and interdisciplinary coordination; however, much of the supporting evidence is observational, and reported associations should not be interpreted as establishing causality. Available studies remain heterogeneous, and direct dental care should not be assumed to be necessary for every hospitalized patient.

A differentiated, risk-based model may therefore be more appropriate than a uniform approach, with the intensity of dental involvement tailored to patient needs, clinical risk, functional dependency, and institutional capacity.

## Introduction and background

Hospitalization can substantially alter a patient’s ability to maintain oral health. Acute illness, functional dependency, cognitive impairment, altered nutrition, xerostomia, reduced mobility, and reliance on caregivers may interfere with normal oral-hygiene routines. Earlier studies have documented substantial oral-health needs among hospitalized adults and have suggested that oral conditions may deteriorate during admission, particularly among patients who are unable to perform effective self-care [1,2].

The clinical relevance of oral health during hospitalization extends beyond local discomfort or oral disease. Impaired oral-health status at admission has been associated with poorer functional recovery, prolonged hospitalization, and mortality in several inpatient populations. Poor oral health may also coexist with malnutrition, dysphagia, frailty, cognitive impairment, and dependence in activities of daily living, making oral status potentially relevant as one component of broader clinical vulnerability [3-5]. However, the existing literature remains fragmented across distinct clinical settings, intervention types, and levels of dental involvement, making it difficult to determine which hospitalized patients may benefit from specific oral-care strategies and when professional dental expertise adds value beyond routine nursing care.

Respiratory complications have received particular attention. Oral biofilm and secretions may act as a reservoir of potentially pathogenic microorganisms that can be aspirated, particularly in patients with dysphagia or impaired airway protection. Consequently, a substantial portion of the hospital oral-care literature has focused on respiratory outcomes across distinct clinical settings. In mechanically ventilated ICU populations, much of the evidence concerns ventilator-associated pneumonia (VAP), whereas studies in non-ventilated hospitalized patients have examined hospital-acquired pneumonia, including non-ventilator hospital-acquired pneumonia. Toothbrushing, antiseptic agents, structured oral-hygiene protocols, staff education, and professional dental care have been evaluated across these populations, with varying findings by clinical setting and intervention type [6-8].

Hospital oral-health management encompasses different levels of care, ranging from patient self-care and routine nursing-led hygiene to dental consultation and direct professional treatment. These approaches serve different clinical purposes and should not be considered interchangeable. The appropriate level of care depends on patient functional status, oral disease burden, clinical risk, ability to perform self-care, and available institutional resources.

Existing evidence syntheses have largely examined individual components of hospital oral health in isolation, including oral-hygiene interventions, pneumonia prevention, or dental-team involvement within particular clinical settings. Less attention has been given to integrating baseline oral-health status, broader clinical outcomes, intervention effectiveness, and differing levels of dental professional involvement across hospitalized adult populations.

This fragmentation raises a practical question: which aspects of hospital oral care can be effectively delivered through standardized nursing care, and when does professional dental involvement provide additional value?

The objective of this narrative review is to synthesize current evidence on the relationship between oral-health status and clinical outcomes in hospitalized adults, evaluate the potential impact of oral-health interventions, and examine the role of dental professionals within interdisciplinary hospital care.

## Review

Search approach and narrative synthesis

A structured narrative review approach was used because the research question encompassed heterogeneous patient populations, oral-health exposures, interventions, professional roles, study designs, and clinical outcomes.

PubMed and Scopus were searched in August 2026 for literature published primarily from 2015 through 2026. Four thematic search strategies were used. Key terms included combinations related to: (1) oral health, oral-health status, hospitalized adults, inpatients, and clinical vulnerability; (2) oral health, hospital-acquired pneumonia, VAP, respiratory infection, bloodstream infection, sepsis, mortality, functional outcomes, and length of stay; (3) oral hygiene, toothbrushing, oral-care protocols, antiseptic agents, hospitalized patients, and hospital-acquired infection; and (4) dentists, dental professionals, hospital dentistry, oral-health teams, interdisciplinary care, and inpatient care. Earlier foundational publications were retained when directly relevant to the clinical or conceptual framework of the review.

Eligible publications were English-language systematic reviews, meta-analyses, randomized controlled trials, quasi-experimental studies, cohort and other observational studies, and clinically relevant intervention studies involving hospitalized adults. Studies were included when they addressed at least one of the review domains: oral-health status, respiratory or broader clinical outcomes, oral-care interventions, or professional dental involvement in hospital care. Studies focused exclusively on pediatric, outpatient, or non-hospital populations were excluded unless they provided essential foundational context. Case reports, editorials, commentaries without primary or synthesized evidence, and non-English publications were excluded.

The searches yielded 734 records in PubMed and 1,127 in Scopus. After deduplication across the four search strategies and both databases using the digital object identifier (DOI) first, PubMed identifier (PMID) when available, and title-year matching when identifiers were unavailable and no conflicting identifier was present, 766 unique records formed the combined search corpus. Titles and abstracts were screened by a single reviewer, and 42 potentially relevant articles were subsequently assessed in full text against the eligibility criteria. Evidence was synthesized narratively and thematically. Because this was a narrative review rather than a systematic review, no formal risk-of-bias instrument or certainty-of-evidence scoring system was applied. Study design, population, intervention characteristics, outcome definition, and major methodological limitations were nevertheless considered during interpretation, with greater interpretive weight given to randomized trials, systematic reviews, and studies directly addressing the review question. No quantitative pooling, meta-analysis, or meta-regression was performed.

Oral health status and clinical vulnerability

Several observational studies suggest that oral-health status at hospital admission may reflect broader clinical vulnerability. In post-acute inpatients, Shiraishi A et al. found that impaired oral health on admission was associated with poorer subsequent clinical and functional outcomes. Among hospitalized older adults, Shyu SW et al. reported associations between severe oral-health impairment, multiple geriatric syndromes, and in-hospital mortality, with severe oral impairment remaining associated with mortality after multivariable adjustment. Maeda K and Mori N similarly reported an association between poor oral-health status and mortality in geriatric inpatients [3-5].

These findings should not be interpreted as evidence that poor oral health independently causes adverse hospital outcomes. Oral impairment frequently occurs among patients who are medically complex, malnourished, frail, cognitively impaired, functionally dependent, or unable to maintain self-care. Nevertheless, oral status may provide clinically relevant information about overall patient vulnerability.

Associations have also been reported across infectious and non-infectious clinical outcomes. In a prospective cohort of critically ill neurological patients, Silveira CF et al. found modest associations between selected oral abnormalities and pneumonia or bloodstream infection, while no oral factor was associated with sepsis. In acute-stroke populations, poorer oral condition has also been associated with hospital-acquired pneumonia and less favorable functional outcomes. Among older adults hospitalized with aspiration pneumonia, poorer oral-health status at admission was independently associated with a longer hospital stay [9-11].

Oral health may further interact with nutritional and functional vulnerability. Yoshimura Y et al. reported that the coexistence of low body mass index and poor oral health among older post-stroke patients was associated with poorer activities of daily living, swallowing function, and cognition. These findings support viewing oral health not in isolation but as one component of a broader clinical profile that may include malnutrition, dysphagia, functional dependency, and frailty [12].

Taken together, these studies support the clinical relevance of oral assessment at admission, while the predominantly observational evidence warrants cautious interpretation.

Oral-care interventions and hospital outcomes

Intervention evidence is strongest for respiratory outcomes, particularly hospital-acquired pneumonia and VAP. In a systematic review and meta-analysis of 15 randomized trials involving 10,742 hospitalized adults, Ehrenzeller S and Klompas M found that daily toothbrushing was associated with a lower incidence of hospital-acquired pneumonia and lower intensive care unit (ICU) mortality. The reduction in pneumonia was statistically significant among mechanically ventilated patients, whereas the estimate in non-ventilated patients was imprecise and did not demonstrate a significant reduction. Reductions in the duration of mechanical ventilation and ICU length of stay were also reported, while several other hospital outcomes were not significantly changed [6].

The 2020 Cochrane review similarly found that some oral-hygiene strategies may reduce VAP among mechanically ventilated patients, although certainty varied according to the intervention evaluated. Chlorhexidine-containing oral care probably reduced VAP incidence without demonstrating a benefit for mortality or duration of mechanical ventilation, while evidence supporting toothbrushing was of lower certainty [7].

Evidence beyond mechanically ventilated ICU populations

Although much of this evidence originated in mechanically ventilated populations, the clinical question extends to patients hospitalized outside the ICU. Earlier ward-based studies suggested potential benefits from structured oral-hygiene programs while also highlighting uncertainty regarding their effect on non-ventilator hospital-acquired pneumonia [13,14].

More recently, White NM et al. reported the HAPPEN multicenter stepped-wedge cluster-randomized trial, which substantially strengthened this evidence base. The trial evaluated 8,870 patients across nine general hospital wards and found that an enhanced multicomponent oral-care program was associated with a lower incidence of non-ventilator hospital-acquired pneumonia than usual care. The intervention incorporated enhanced oral-care delivery and products, patient and staff education, and audit-feedback mechanisms and was delivered by dedicated research nurses. Protocol completion also increased substantially during the intervention period. Importantly, benefit was not demonstrated across all secondary infectious outcomes [15].

The favorable HAPPEN findings, however, should not be interpreted as evidence that all oral-care components or protocols produce equivalent effects. Results for individual oral-care components remain less consistent. Evidence regarding chlorhexidine remains inconsistent across contemporary syntheses. Some systematic reviews have reported reductions in VAP incidence, whereas a 2024 network meta-analysis found no significant advantage for chlorhexidine at the evaluated concentrations. Differences in trial selection, intervention protocols, comparator groups, and analytic approaches may contribute to these divergent conclusions [7,16,17].

A 2026 systematic review of heterogeneous ICU oral-hygiene protocols likewise found little consistent evidence of reductions in VAP or mortality across the included studies. The review also highlighted variation in intervention content, personnel, and reporting of baseline dental conditions [18].

Observational evidence has additionally explored routine oral care and patient-directed self-care. Kluberg SA et al. reported associations between persistent routine oral care and lower rates of non-ventilator hospital-acquired pneumonia, while Nishi H et al. found that a hospital-based oral self-care promotion program was associated with lower rates of pneumonia and other infections after adjustment. These associations remain subject to residual confounding because of their observational designs [19,20].

Overall, the intervention literature supports a potential respiratory benefit from structured oral care in selected hospitalized populations, although the magnitude and consistency of benefit vary according to population, intervention components, implementation fidelity, and the outcome evaluated.

What does professional dental involvement add?

The evidence supporting direct professional dental involvement is smaller than the evidence for routine oral-hygiene interventions, but several studies suggest potential additional value.

One of the most direct evaluations comes from Bellissimo-Rodrigues WT et al., who randomized 254 ICU patients to routine oral care alone or routine care supplemented by professional dental treatment. Lower respiratory tract infections occurred less frequently in the dental-intervention group, whereas mortality did not differ significantly between groups. A related publication from the same randomized population reported improvements in oral and gingival outcomes. These publications should therefore be interpreted as complementary reports from one randomized trial rather than as independent trials [21,22]. Because this randomized trial was conducted specifically in ICU patients, its findings should not be extrapolated as evidence supporting routine dentist presence on general hospital wards.

Systematic evidence remains limited. de Araújo EC et al. reported a lower incidence of VAP among ICU patients receiving care involving oral-health teams, but the certainty of evidence was very low, and no significant mortality benefit was demonstrated [23]. Evidence from nonrandomized studies should be interpreted separately from randomized trial evidence because these designs are more susceptible to confounding and cannot establish causal effects with the same degree of certainty.

Nonrandomized evidence and interpretation

Quasi-experimental evidence also suggests potential benefit but requires cautious interpretation. Ribeiro IL et al. reported that implementation of repeated dentist-provided oral hygiene and periodontal care was associated with lower ICU mortality compared with preceding periods, whereas VAP incidence density did not significantly change. Because the comparison was nonrandomized and temporal, causal attribution remains uncertain [24].

Evidence outside traditional ICU populations is emerging. Among 1,415 older adults hospitalized with acute pneumonia, Ohi T et al. found that professional oral-health management by hospital dentistry was independently associated with greater odds of favorable discharge. Professional management could include mechanical cleaning, periodontal treatment, restorative treatment, denture adjustment, or extraction according to patient needs. However, treatment allocation was observational rather than randomized, and residual confounding cannot be excluded [25].

The available evidence is more consistent with a differentiated rather than universal model of dental involvement, in which trained nursing staff may effectively deliver routine oral care, while dental professionals provide additional expertise when assessment, diagnosis, or treatment extends beyond bedside hygiene.

Models of dental integration in hospital care

Dental expertise can enter hospital care through several distinct models rather than through a single standardized approach.

At one end of this continuum, patients may perform oral self-care when clinically able, or routine oral hygiene may be delivered by nursing staff. Nursing-led oral care remains a fundamental component of hospital care. Practice-guideline development has specifically addressed oral care for care-dependent hospitalized adults outside the ICU [26], while intervention evidence shows that structured bedside programs can improve care delivery and, in selected settings, respiratory outcomes [15].

A second model involves dental-supported nursing care, in which nurses remain responsible for routine bedside oral hygiene but receive training, protocols, or support from dental professionals. Suzuki H et al. compared oral-health management delivered directly by dental professionals with management delivered by nurses who had received instruction from dental professionals. Both groups improved in overall oral-health status, while direct professional care produced additional improvement in some denture-related findings. These results suggest that direct dental-professional care may not be necessary for every component of inpatient oral management [27].

A third model is a dentist-led oral-care system. Ozaki K et al. described a systems-level approach in which a full-time dentist provided staff education, practical training, oral assessment, standardized oral-care procedures, and pathways for nurse-initiated dental referral. Pneumonia incidence was lower after implementation of the dental-care system, although the retrospective comparison across different time periods limits causal attribution [28].

A fourth model involves an embedded dental professional functioning as part of the inpatient medical team. Simon L et al. demonstrated the feasibility of integrating a dental hygienist into an inpatient team caring for patients with diabetes. The model was feasible and well accepted, although the study was not designed to demonstrate improvement in major clinical outcomes [29].

Finally, some hospitalized patients may require direct professional dental treatment. This may include diagnosis and management of active oral disease or other problems beyond routine bedside care. Bellissimo-Rodrigues WT et al. provide the clearest randomized example of this more intensive model, while observational and quasi-experimental studies illustrate additional applications of professional dental care [21,24,25].

Prospective observations in hospitalized stroke patients further demonstrate that referral to dental services can improve oral-health measures during acute and subacute hospitalization, although these studies have primarily evaluated oral rather than systemic clinical outcomes [30].

Taken together, these models suggest that the role of dental professionals in hospital care is better understood as a spectrum of involvement tailored to patient needs and institutional capacity rather than as a single uniform intervention.

To organize these patterns, we propose a conceptual continuum ranging from routine nurse-led oral care to direct professional dental treatment (Figure 1).

**Figure 1:**
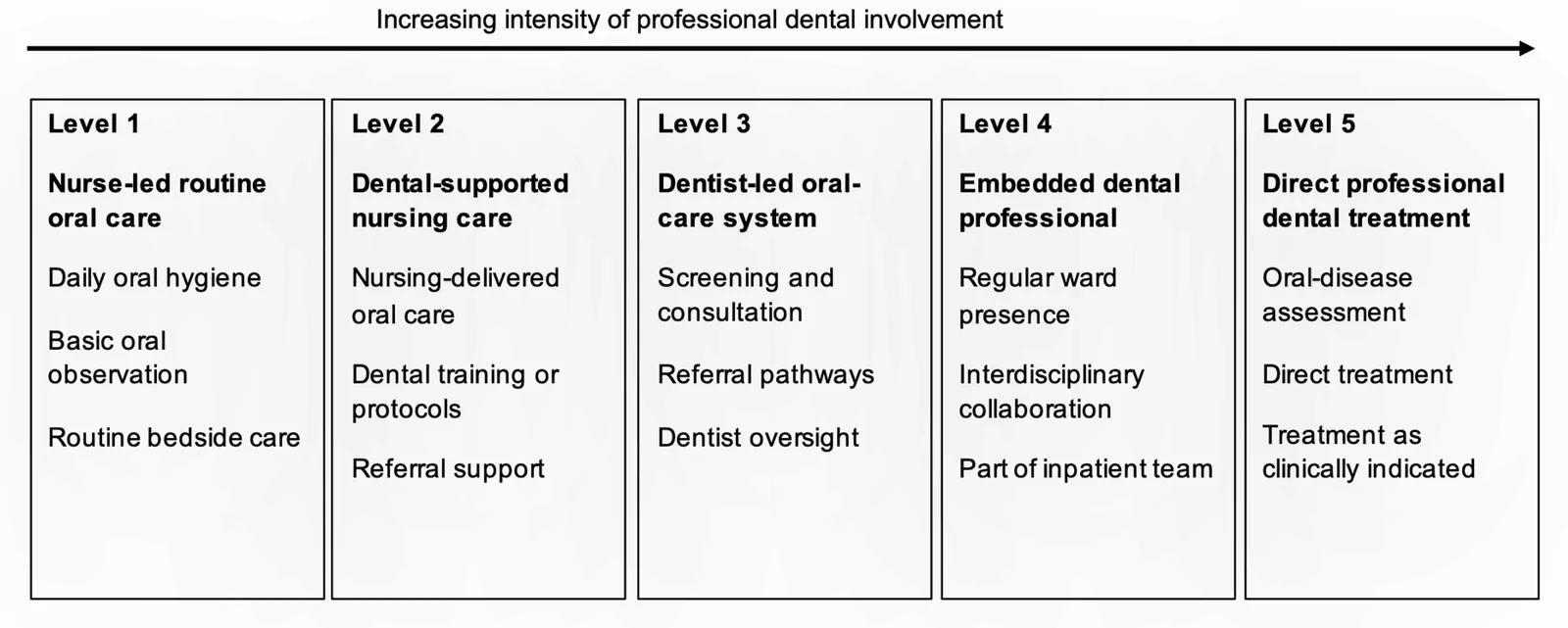
Conceptual continuum of dental involvement in hospital care. Author-developed conceptual framework. The levels represent increasing intensity of professional dental involvement and do not imply superiority, a mandatory sequence, or a validated clinical hierarchy. The appropriate level may vary according to patient needs and institutional resources. Figure created by the author using Microsoft Word and Microsoft PowerPoint.

This continuum is an author-developed organizational framework and has not been clinically validated. The levels represent increasing intensity of professional dental involvement rather than increasing quality of care and should not be interpreted as a mandatory sequence. The appropriate level may vary according to oral-disease burden, functional dependency, clinical risk, patient capacity for self-care, available personnel, and institutional resources. Representative studies informing the narrative synthesis across these domains are summarized in Table 1.

**Table 1:** Representative evidence on oral health, oral-care interventions, dental involvement, and hospital outcomes. HAP: Hospital-acquired pneumonia; RCT: Randomized controlled trial; VAP: Ventilator-associated pneumonia. Source: Original table created by the author based on the studies included in this narrative review.

Study	Design/Population	Exposure or Intervention	Main Outcome(s)	Principal Caution
Shiraishi A et al. [3]	Prospective cohort; post-acute inpatients	Oral-health status assessed at admission	Poorer clinical and functional outcomes associated with impaired oral health	Observational design; does not establish causality
Shyu SW et al. [4]	Retrospective observational study; 1,141 hospitalized adults ≥65 years	Severity of oral-health impairment	Associated with geriatric syndromes and in-hospital mortality	Residual confounding possible
Silveira CF et al. [9]	Prospective cohort; 248 critically ill neurological patients	Oral abnormalities assessed during ICU admission	Selected oral findings associated with pneumonia or bloodstream infection	Modest associations; no oral factor associated with sepsis
Ehrenzeller S and Klompas M [6]	Systematic review and meta-analysis; 15 RCTs, 10,742 hospitalized adults	Daily toothbrushing	Lower HAP incidence and ICU mortality; shorter duration of mechanical ventilation and ICU stay	Pneumonia benefit clearest in mechanically ventilated patients; non-ventilated estimate imprecise
White NM et al. (HAPPEN) [15]	Multicenter stepped-wedge cluster-randomized trial; 8,870 patients across nine hospital wards	Enhanced multicomponent oral-care program	Lower incidence of non-ventilator hospital-acquired pneumonia	Multicomponent intervention delivered by dedicated research nurses; benefit not shown across all secondary infectious outcomes
Bellissimo-Rodrigues WT et al. [21,22]	Randomized clinical trial; 254 ICU patients	Routine nursing oral care with or without additional professional dental treatment	Lower respiratory tract infections and improved oral/gingival outcomes; mortality unchanged	2014 and 2018 reports describe the same randomized patient population
de Araújo EC et al. [23]	Systematic review; five ICU studies	Care involving oral-health teams	Lower VAP incidence; no significant mortality benefit	Very low certainty of evidence
Ribeiro IL et al. [24]	Quasi-experimental study; critically ill ICU patients	Dentist-provided oral hygiene and periodontal treatment at least three times weekly	Lower ICU mortality during the intervention period; VAP incidence density not significantly changed	Nonrandomized temporal comparison limits causal inference
Ohi T et al. [25]	Observational cohort; 1,415 older adults hospitalized with acute pneumonia	Professional oral-health management by hospital dentistry	Associated with greater odds of favorable discharge	Observational treatment allocation; residual confounding possible
Ozaki K et al. [28]	Retrospective cohort; 945 patients with acute stroke	Dentist-led oral-care system with staff education, assessment, standardized care, and referral pathways	Lower pneumonia incidence after system implementation	Pre-, during-, and post-implementation periods introduce temporal confounding
Suzuki H et al. [27]	Longitudinal study; 117 hospitalized patients receiving Nutrition Support Team care	Oral-health management by dental professionals versus nurses trained by dental professionals	Overall oral-health status improved in both groups; additional denture-related improvement with dental professionals	Evaluated oral-health outcomes rather than major systemic clinical outcomes
Gomes RF et al. [18]	Systematic review; 23 ICU studies	Heterogeneous oral-hygiene protocols	Little consistent evidence of reductions in VAP or mortality across protocols	Substantial heterogeneity in interventions, personnel, and reporting of baseline dental conditions

Implementation challenges and remaining evidence gaps

Implementation of inpatient oral care depends on more than the selection of an effective clinical protocol. Barriers occur at the patient, provider, and organizational levels. Qualitative studies have identified limited confidence, perceived procedural risk, time pressure, competing clinical priorities, uncertainty regarding professional responsibility, and insufficient resources as barriers to oral-care delivery. Implementation projects have further shown that improvements in staff awareness and capacity do not necessarily translate into measurable changes in multidisciplinary practice or patient outcomes [31,32].

Recent scoping reviews by Pojmonpiti S et al. similarly highlight the importance of staff training, availability of oral-care products, staffing, interdisciplinary collaboration, institutional support, audit systems, provider prioritization, and patient-specific limitations [33,34]. Survey evidence from ICU nursing staff also suggests that dental-professional presence, staff training, and institutional protocols may influence oral-care practice [35]. These findings suggest that implementation should be considered an active component of hospital oral-health interventions rather than a background condition. An intervention that is effective under research conditions may have limited impact when bedside delivery is inconsistent or when referral pathways for conditions beyond routine care are unavailable [33,34].

Interpretation is further complicated by heterogeneity in populations, intervention components, and outcome definitions. Studies range from mechanically ventilated ICU patients to stroke, geriatric, rehabilitation, pneumonia, and general medical populations, while “oral care” may encompass interventions as different as toothbrushing, antiseptics, denture care, staff education, or professional dental treatment.

This heterogeneity limits direct comparison across studies and complicates the identification of patients most likely to benefit from each approach.

Consequently, the central unresolved question is not simply whether oral care is beneficial, but which hospitalized patients require which components and levels of oral-health management, and when dental expertise adds value beyond standardized bedside care.

Future directions

Future research should prioritize patient selection, attribution of intervention components, the incremental value of dental expertise, and implementation fidelity. Patients with substantial oral disease, dysphagia, neurological impairment, prolonged hospitalization, severe functional dependency, complex prostheses, or an inability to perform self-care may have different needs from medically stable patients who can maintain their own oral hygiene. At the same time, intervention components such as toothbrushing, antiseptic use, moisturization, denture care, oral assessment, staff education, referral systems, and dental treatment should be described separately whenever possible so that the contribution of each component can be evaluated.

Trials evaluating professional dental involvement should examine its incremental value beyond standardized nursing oral care. Comparisons between nursing care alone and nursing care supplemented by dental assessment, consultation, or treatment would help determine which aspects genuinely require dental expertise. Implementation should also be measured directly, including adherence, training, staffing, referral completion, access to oral-care products, patient tolerance, and other factors that influence whether an intervention is actually delivered as intended.

The key unanswered question is therefore: Which hospitalized patients benefit from dental-professional involvement, at what level of intensity, and which dental-specific components provide benefit beyond standardized nurse-delivered oral care? Answering this question could help move hospital oral-health management from a uniform approach toward a more individualized, risk-based model of interdisciplinary care.

## Conclusions

Poor oral health is associated with clinical vulnerability and adverse outcomes in hospitalized adults, although much of the evidence is observational. Structured oral-care interventions have the clearest support for respiratory outcomes, with recent randomized evidence extending beyond mechanically ventilated ICU populations to non-ventilated patients in general hospital wards.

Professional dental involvement may add value through assessment, diagnosis, treatment of active oral disease, staff education, and interdisciplinary coordination, but current evidence does not support a universal level of dental involvement for all hospitalized patients. A differentiated, risk-based approach may therefore be appropriate, ranging from routine nurse-led oral care to direct professional dental treatment according to patient needs and institutional capacity. Future research should determine which patients benefit from each level of involvement and which dental-specific components provide value beyond standardized nursing oral care.
